# Tracked Physical Activity Levels Before and After a Change in Incentive Strategy Among UK Adults Using a Rewards App: Retrospective Quasi-Experimental Study

**DOI:** 10.2196/50041

**Published:** 2024-12-10

**Authors:** Hannah McCarthy, Henry W W Potts, Abigail Fisher

**Affiliations:** 1 University College London London United Kingdom

**Keywords:** mHealth, rewards, incentives, physical activity, smartphone, apps, mobile apps, app-based intervention, behavior change, exercise

## Abstract

**Background:**

Financial incentives delivered via apps appear to be effective in encouraging physical activity. However, the literature on different incentive strategies is limited, and the question remains whether financial incentives offer a cost-effective intervention that could be funded at the population level.

**Objective:**

This study aimed to explore patterns of tracked physical activity by users of an incentive-based app before and after a change in incentive strategy. A business decision to alter the incentives in a commercially available app offered a natural experiment to explore GPS-tracked data in a retrospective, quasi-experimental study. The purpose of this exploratory analysis was to inform the design of future controlled trials of incentives delivered via an app to optimize their usability and cost-effectiveness.

**Methods:**

Weekly minutes of tracked physical activity were explored among a sample of 1666 participants. A Friedman test was used to determine differences in physical activity before and after the change in incentive strategies. Post hoc Wilcoxon tests were used to assess minutes of physical activity in the 2 weeks before and after the change. A secondary analysis explored longitudinal patterns of physical activity by plotting the mean and median minutes of physical activity from 17 weeks before and 13 weeks after the change in incentive strategy. CIs were calculated using bias-corrected bootstraps. Demographics were also explored in this way.

**Results:**

There were significant differences in the weekly minutes of activity before and after the change in incentive strategy (Friedman χ^2^_2_=42, *P*<.001). However, a longitudinal view of the data showed a more complex and marked variation in activity over time that undermined the conclusions of the before/after analysis.

**Conclusions:**

Short-term before-and-after observational studies of app-tracked physical activity may result in misleading conclusions about the effectiveness of incentive strategies. Longitudinal views of the data show that important fluctuations are occurring over time. Future studies of app-tracked physical activity should explore such variations by using longitudinal analyses and accounting for possible moderating variables to better understand what an effective incentive might be, for whom, and at what cost.

## Introduction

It is well established that sufficient physical activity is important for good health. Despite this, the World Health Organization still reports that 1 in 4 adults and 2 in 4 adolescents are inactive, with a global direct cost of $54 billion and $14 billion attributed to lost productivity [1]. Over 4 billion people currently use smartphones worldwide, with this number projected to reach 6.1 billion by 2029 [2]. With such widespread use, mobile apps show promise as delivery channels for incentives that promote physical activity [3].

Systematic reviews of randomized controlled trials and observational real-world studies suggest that financial incentives are effective in encouraging physical activity [4-8]. Despite their apparent effectiveness, the question remains as to whether financial incentives can offer a cost-effective intervention that should be funded at a population level [9]. A Canadian incentive program appeared to be cost-effective as the reward levels were kept at a minimum, but it was closed due to funding constraints [10]. A longitudinal study that reduced reward size over time in the same Canadian intervention found that the number of points (or size of the reward) offered was not as important as the type, timing, and content [4]. Vitality UK is a health and life insurance company that offers Vitality Active Rewards, a reward program for staying active. Vitality’s Active Rewards program was the subject of an observational real-world study in 2019. The researchers found that larger long-term incentives alone did not increase physical activity or prolong engagement as effectively as when combined with smaller immediate incentives. Another UK study using the Sweatcoin app, which converts steps into a digital currency that can be exchanged for vouchers, looked at the mean daily step count 3 months after registering on the app compared with the mean daily step count in each of the 3 months before registration. They found that the step count increased significantly over the 6-month period [7].

While real-world incentive-driven interventions show promise, there remains a question about the feasibility of financial incentives in the long term [11,12]. This has led to calls for research that systematically manipulates reward size and win probabilities to explore methods for reducing costs [13]. Lottery-based incentives, rather than assured rewards, may offer a way to control costs by fixing the payout [14-22].

In Singapore, a 2021 study of more than 1 million people employed small-scale, immediate, or lottery incentives and observed increased daily steps in users [8]. The researchers used real-time data analytics to inform their incentive strategy and other design features of the intervention, which varied over 3 waves. However, there is little evidence of the relative effectiveness of lottery-based incentives compared with assured rewards. To our knowledge, Patel and colleagues [17,18] are the only researchers who have directly compared lottery and assured incentives in an app-based study. They compared daily, assured, gain-based incentives, lottery incentives, and loss incentives, ultimately finding that only loss-framed incentives were effective compared with control [18]. They also compared different lottery conditions—higher frequency smaller reward, jackpot, and combined—and found only the combined lottery incentive to be significantly greater than the control [17]. In another study where participants were given the choice to receive rewards as guaranteed cash payments or a lottery ticket with a 1-in-10 chance of receiving the same expected value, not surprisingly, most opted for the guaranteed payout; therefore, the lottery-group sample size was inadequate to make a meaningful comparison [20].

In commercial app-based interventions, the incentives are often subject to change, usually because of a business or financial decision rather than being theory-based or driven by research aims [23,24]. Changes in app incentive features offer the opportunity to observe patterns in data before and after the change, giving insights into which ones may be worthy of further analysis or study [25]. Observational studies generate a lot of ecologically valid data, but the lack of randomization between treatment conditions means that it is not possible to draw conclusions about causation because other confounding variables may be driving the outcomes [26].

This study aimed to explore patterns of tracked physical activity by users of a commercial app before and after a change in incentive strategy. This is a quasi-experimental study that took advantage of a business decision to alter the incentives in the BetterPoints app from certain points to lottery-focused to control costs. A secondary aim was to better understand how to analyze app-derived data sets to lay the groundwork for future trials that may help to establish the causal effects of different incentive strategies.

## Methods

### Overview

This study was a retrospective exploration of weekly minutes of tracked physical activity data collected by users of the commercially available app BetterPoints, before and after a change in incentives. Data collected between May and December 2018 were used.

### Participants

Participants were not recruited to the study as it used data that had been collected in the normal course of delivering the app. An initial SQL database query was run on the BetterPoints server to extract anonymous IDs and basic demographics for all users who were not in any sponsored BetterPoints program (to control for other bonus incentives that have been present in sponsored programs). Data from app users who registered after the start of the study period or who tracked nothing during the whole study period were excluded. Demographic data were collected as part of the standard registration process on the BetterPoints app, including year of birth, gender, and postal code. Indices of multiple deprivation (IMD) were derived from postal code data using the Ministry of Housing Communities and Local Government online postal code lookup tool. Descriptive statistics were run for age, gender, IMD, physical activity levels at baseline, and time registered on the app, as shown in the Results section.

### Ethical Considerations

Consent for research was provided by the BetterPoint users upon registration and acceptance of terms and conditions. All data were anonymized prior to analysis. Ethical approval was received from the University College London (UCL) Research Ethics Committee for the project (19279/001).

### Variables

This study’s main outcome variable was weekly minutes of app-tracked physical activity during periods with exposure to different incentive strategies. Demographic variables were explored as possible moderators.

Users of the app are rewarded for tracked physical activity with a digital currency called “BetterPoints.” Along with points that can be exchanged for vouchers in the app, it is possible to reward physical activity with lottery tickets for prize draws, known as “BetterTickets,” which can be set up to be drawn automatically every day, week, or month. Prizes may be in the form of a “pot” of BetterPoints or specific predefined prizes such as cinema tickets.

Points are an assured token currency that has a financial value. In this study, 1000 points were equivalent to £1 (US $1.32 at the time of writing). They can be accrued over time and then exchanged for vouchers in a reward catalog within the app. Rewarding users with assured points scales linearly with the number of people (ie, the higher the number of people, the higher the cost). In a lottery-based incentive schedule, the prize draw tickets that participants earn for doing physical activity do not have a direct cost associated with each ticket, the cost of the prizes can be fixed, and the cost will not increase with the number of tickets issued. However, the expected gain of a ticket will be reduced with more users if costs are kept constant.

The decision was made by BetterPoints Ltd to continue to give participants the chance to earn points and tickets for physical activity but to reduce the reliance on points to control the budget as user numbers increased. From September 1, 2018, onward, there was less daily emphasis on points and more emphasis on lottery tickets. This meant that the incentive strategy still combined points and lottery tickets but with no point-based incentives for daily physical activity after August 31, 2018. The 2 periods with different emphasis on points versus lottery strategies are referred to as before the change (May-August 2018) and after the change (September-December 2018). Before, participants could earn 1 point per minute for up to 30 minutes of activity per day. Afterward, participants only received tickets into a monthly prize draw for their daily activity. The stretch goal of 150 minutes per week for 4 or more weeks was also incentivized differently. Here, the emphasis was reversed: before the change, participants only got lottery tickets for a stretch goal, while after the change, participants were able to earn 1000 points if they met the stretch goal. In both challenges, the lottery prizes were all pots of BetterPoints that could then be “spent” on vouchers in the app catalog. These incentive strategies are detailed in Table 1. App users were informed of the changes via messages on their app homepage.

**Table 1:** Details of change in incentive strategies^a^.

Incentive feature changed			Before		After
**Goal**					
	Daily	30 minutes in two 15-minute increments		30 minutes in three 10-minute increments and stretch goal of 30 minutes in one go	
	Weekly	150 minutes		150 minutes	
	Monthly	150 minutes in consecutive weeks		150 minutes in consecutive weeks	
**Certainty**					
	Daily	Certain (points)		Uncertain chance (lottery)	
	Weekly	Uncertain chance (lottery)		Uncertain chance (lottery)	
	Monthly	End of challenge uncertain chance (“Jackpot” lottery)		Uncertain chance and Certain (points)	
**Incentive amount**					
	Daily	1 point per minute up to 30 points a day		1 ticket per 10 minutes up to 3 times a day. Stretch bonus 30 tickets for monthly draw	
	Weekly	2 tickets per 20 minutes		150 tickets for monthly prize draw	
	Monthly	1 ticket per 150 mins a week		150 tickets per 150 mins a week up to 600 tickets in the month. 1000 Points for stretch goal	
**Maximum reward value**					
	Daily	30 points worth 3 p		Monthly prizes worth a total of £180	
	Weekly	15 prizes worth £10 each		Monthly prizes worth a total of £180	
	Monthly	£100 for the user and £100 for charity		Monthly prizes worth a total of £180 plus 1000 points bonus worth £1	

^a^A currency exchange rate of GPB £1=US $1.32 applies.

### Data Sources and Treatment

Weekly minutes of physical activity were collected from BetterPoints app users via GPS sensors in their smartphones. Both Android and iOS handsets can run the app. Users can turn on automatic tracking, which collects data on physical activity, automatically classifies it, and detects when movement has stopped. At the time of the study, users could also use manual tracking, which allowed them to select a particular type of activity, such as walking or cycling, from a menu of options. Manual tracking required the user to complete the activity when they no longer wished to track.

On some days, the app would record no physical activity for a user. The way the app tracks activity means that an activity like moving around the house is not recorded. On these occasions we could not be certain of what happened in the user’s life: they may have had a largely inactive day, but they may also have been active without their phone, or they may have turned off automatic activity tracking and omitted to manually track activity they took. We chose to assume that these were genuine cases with minimal activity and recorded them as 0 minutes of physical activity. The statistical distribution of the activity was largely unchanged by this decision, so we are confident that this imputation did not bias the results.

### Study Size

The study size was determined based on the available data and the eligibility criteria described earlier (ie, people who had registered at the start of the study and tracked at least 1 activity during the study period).

### Statistical Tests

As the tracked minutes of physical activity data per week were highly skewed, a nonparametric Friedman test was conducted, followed by post hoc Wilcoxon tests to assess the difference in weekly minutes of physical activity. Time periods were defined as follows; week commencing August 18 (T1), August 25 (T2), September 1 (T3), and September 8 (T4). Pairwise comparisons were tested.

The initial analysis plan for this study was predicated on a before and after design. We subsequently explored the data further and presented numerous exploratory plots. Mean, SD, and median minutes of physical activity were plotted from 1 month before the start of the assured points condition to the end of the lottery condition (from the week starting May 5, 2018, to the week starting November 24, 2018). CIs were calculated using bias-corrected bootstraps. This analysis undermined our statistical tests, so further exploratory analyses were conducted for age, gender, and IMD band.

## Results

A total of 1666 app users were registered on or before the start of the study and had tracked an activity during the study period. These users were 53% (n=881) female, with a mean age of 42 (SD 12.13) years. Among them, 29.6% (n=493) were from the most deprived deciles. Table 2 summarizes demographic data of participants.

The mean weekly minutes of physical activity were 137 (SD 203) at T1, 138 (SD 209) at T2, 156 (SD 219) at T3, and 153 (SD 206) at T4.

Table 3 shows the mean, SD, and median for each time point.

Overall, there were significant differences in the weekly minutes of activity before and after the change in incentive strategy as assessed by a Friedman test (χ^2^_2_=42, *P*<.001).

The results of post hoc Wilcoxon signed-rank tests indicated a significant difference in the paired observations between the last week of the assured points incentive condition (T2: mean 138, SD 203) and the first week of the lottery incentive condition (T3: mean 156, SD 219) with a *z* score of –4.14 (**P*<.001*). Table 4 shows the results of all the pairwise comparisons.

**Table 2:** Participants’ demographic data (N=1666).

Characteristic		Participants	
Age (years), mean (SD)		42 (12.13)	
Missing, n (%)		327 (19.62)	
**Age (years), n (%)**			
	14-24		103 (6.2)
	25-34		292 (17.5)
	35-44		409 (24.5)
	45-54		336 (20.2)
	55-64		153 (9.2)
	65+		46 (2.7)
**Gender, n (%)**			
	Missing		256 (15.4)
	Male		523 (31.4)
	Female		881 (52.9)
	Other		6 (0.4)
**Indices of multiple deprivation, n (%)**			
	Missing		57 (3.4)
	1-3 (most deprived)		493 (29.6)
	4-7		652 (39.1)
	8-10 (least deprived)		464 (27.9)

**Table 3:** Mean and median weekly minutes of physical activity at different time points.

Time point	Mean (SD)	Median
T1	136.64 (203)	44
T2	137.72 (209)	52
T3	155.84 (219)	71
T4	153.09 (206)	64

**Table 4:** Post hoc comparison of weekly minutes of physical activity before and after the change in incentive strategies.

Comparison weeks	*z* score	*P* value
T2 cf^a^T3	–4.14	<.001
T1 cf T3	–5.71	<.001
T2 cf T4	–5.06	<.001
T1 cf T4	–3.64	<.001
T3 cf T4	0	.87
T1 cf T2	–1	.27

^a^cf: confer (Latin), meaning to compare.

**Figure 1:**
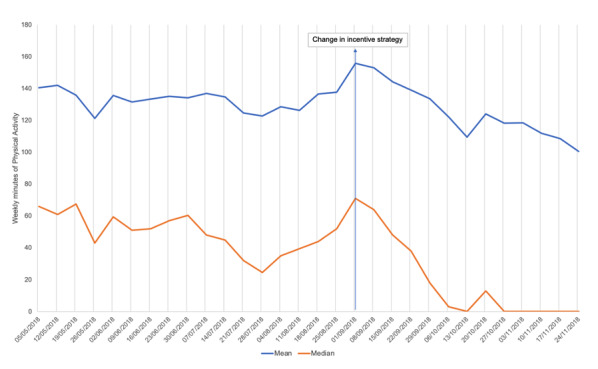
Mean and median weekly minutes of physical activity.

**Figure 2:**
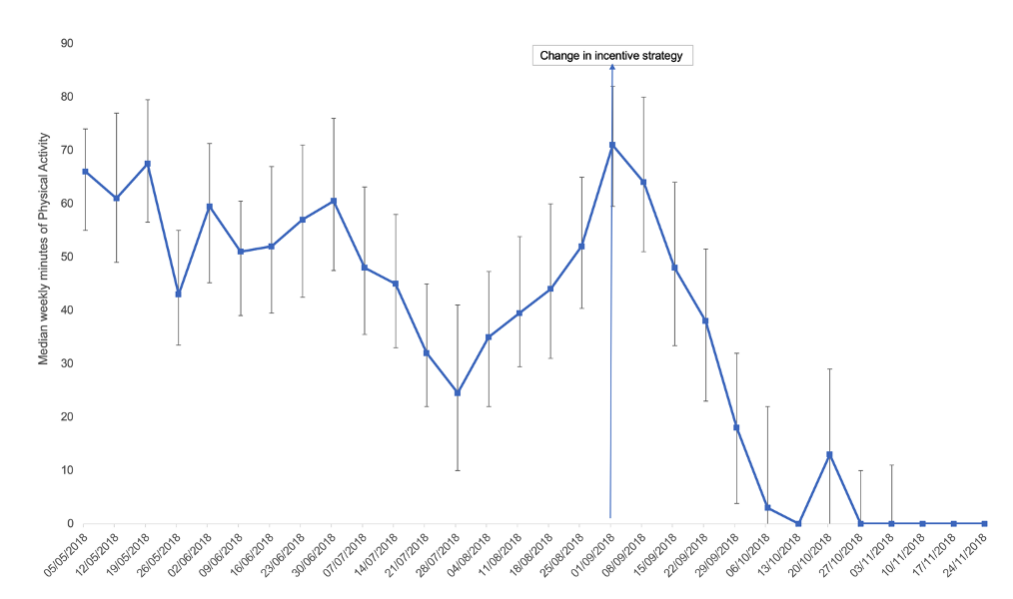
Median weekly minutes of physical activity with CIs.

Secondary analyses explored physical activity over time. Plots are presented in Figures 1 and 2 show a more complex picture than before/after comparisons. They suggest app users were waning in their activity levels during the middle part of the incentive schedule, before the change, and increased their physical activity in the lead-up to the change in incentives. There was a peak in activity at the time of the change in incentives strategy, and then physical activity levels dropped quite dramatically. The median weekly activity reached 0 in the week commencing October 27, 2018, and remained there.

CIs overlapped during the period before and after the change in incentives. Note that the CIs were derived from bootstrapped simulations within each week and are not as powerful as the within-subject statistical tests.

**Figure 3:**
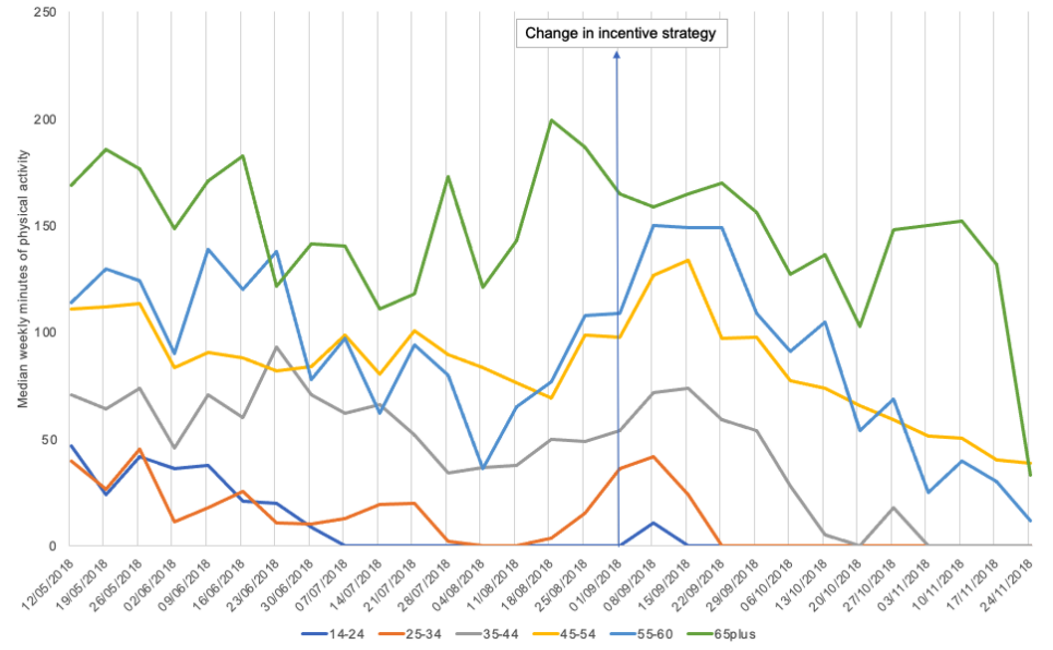
Median weekly minutes of physical activity by age.

**Figure 4:**
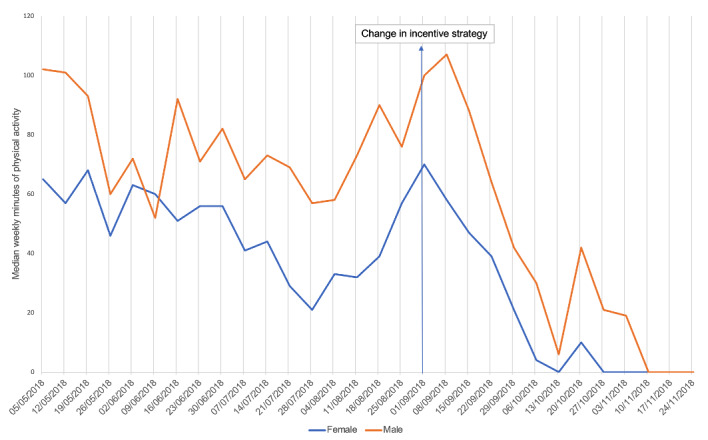
Median weekly minutes of physical activity by gender.

**Figure 5:**
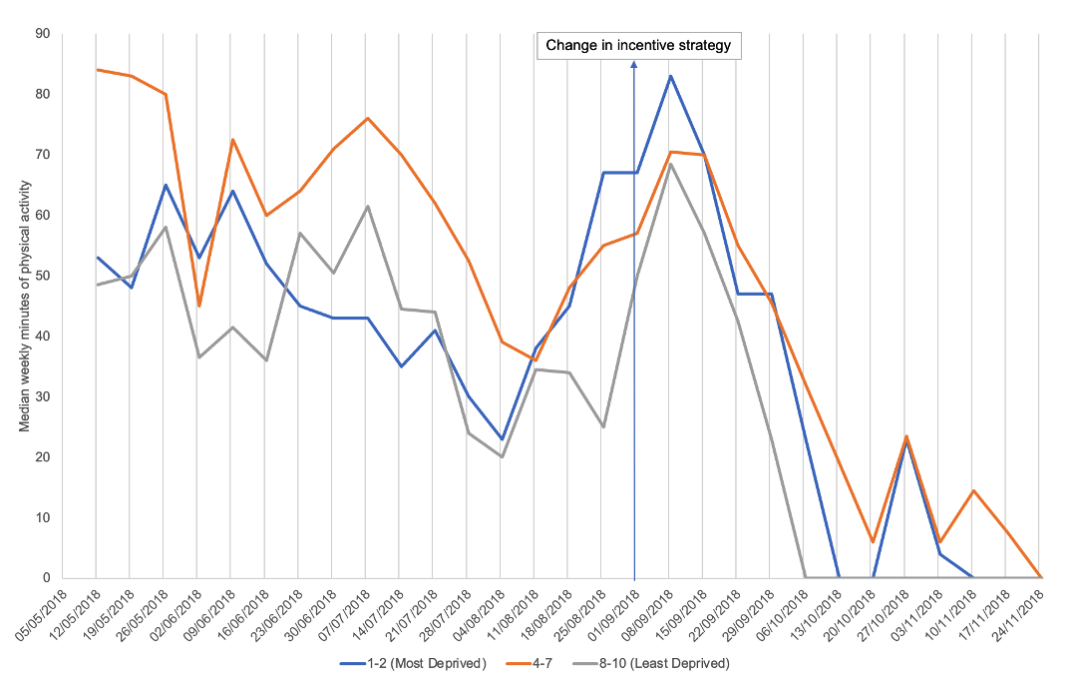
Median weekly minutes of physical activity by indices of multiple deprivation (IMD) decile.

The plot in Figure 3 shows differences in the variation of tracked physical activity around the time of the change in incentives. This suggests that age may moderate response to different incentive designs. The plots in Figures 4 and 5 show that men and women and those in different IMD deciles tracked similar patterns of physical activity over time, which may indicate that gender and socioeconomic status are less important moderators.

## Discussion

This study aimed to explore patterns of tracked physical activity by users of a commercial app before and after a change in incentive strategy. The last 2 weeks before the change and the first 2 weeks after the change were statistically significantly different, suggesting that the change in incentives to a greater focus on lottery awards led to more physical activity among users. However, time-series analysis with CIs shows that important fluctuations occurred over time in the tracked physical activity data which require further study to explain. This suggests the significant before/after difference may not represent the effect of a change in incentive strategies. We note that had we not carried out the longitudinal analysis, we may have been misled by the statistical tests to draw premature conclusions about the relative effectiveness of assured versus lottery-based incentive strategies.

It may be possible that individual differences are moderating the effectiveness of incentive strategies over time. We conducted some exploratory analysis, but further research is needed on demographic and psychological factors that may be moderating responses to incentives. Age may be a factor, as the plots showed that those in different age groups had different patterns in their tracked weekly minutes of physical activity around the time of the change in incentives. Men and women and those in different IMD deciles showed similar variation in physical activity over time, suggesting that they may be responding in a similar way to the change in incentives. The potential for demographics, personality, and prior behavior to moderate the effectiveness of incentives on physical activity outcomes was beyond the scope of this paper and requires further study.

This study highlights the difficulties and limitations of using real-world data in a retrospective study. When the study was originally conceived, the change in incentive strategy seemed straightforward: more emphasis on the lottery and less on assured points. However, upon closer inspection, the complexity of the 2 strategies became apparent, as shown in Table 1. Multiple changes to different features were made simultaneously, including the goals, certainty, and incentive amount. It was impossible to ascertain retrospectively if the actual value of the reward in monetary terms equalized due to the unknown total sample for whom the rewards were available (ie, those in sponsored programs) and the multiple available prizes that would have made calculating the expected value difficult. Future work should simplify incentive strategies and equalize value across assured points and lottery-based strategies.

The continuous nature of real-world data, with individuals regularly joining and leaving the app, required decisions to be made over what data to include in the analysis. We chose to remove users who joined after the study start date and/or tracked nothing during the study, but these approaches could be reconsidered in future studies. Patterns of engagement with an app are also important.

The quasi-experimental nature of this study does not allow for conclusions regarding causality and the relative effectiveness of different incentive strategies [26]. Before and after tests appear, at best, inadequate for assessing the effectiveness of incentive strategies and, at worst, misleading. The method of statistical process control that has recently moved from manufacturing to health informatics may be informative here. Health data are often not in control/stable, which means they do not lend themselves well to before-and-after studies because we do not know if deviations in observed health behaviors are correlated with changes introduced, just part of their natural fluctuation, or the result of some other confounding or moderating factors. The challenge for this area of research is how to transfer more robust methods to real-world settings.

Fluctuations in tracked physical activity over time have typically been given less attention than before and after results derived from static tests of difference at set time points. App-based studies using the Carrot rewards app in Canada and the Vitality app in the United Kingdom both suggested short-term smaller rewards were more effective; however, both studies also showed large ongoing fluctuations in longitudinal plots [4,6]. Brief explanations pertaining to dips during the December holiday period or seasonality relating to weather were offered but without further analysis. Chew and colleagues [8] in Singapore presented times-series plots of daily step counts in each of the 3 waves, showing that the data were unstable. The researchers were aware of these fluctuations and offered possible explanations relating to external factors such as festival times. They also used these observed changes in daily step counts to prompt changes in the intervention, such as spreading out rewards and the introduction of thematic challenges [8]. However, they did not highlight these variations as possibly undermining conclusions drawn from the analysis of a static baseline before the condition with static follow-up time points. The mean daily step count in the after or postintervention condition could have obscured important weekly variations in the data.

It is not recommended that the before-and-after approach be generalized to other studies that seek to understand the effectiveness of incentives. Future work may look in more detail at fluctuations in physical activity over time. This may employ visualizations, growth modeling, and cluster analysis to understand individual trajectories of physical activity and similarities among app users. Individual psychological, sociodemographic, and behavioral factors that correlate with or predict physical activity trajectories or group memberships should also be examined. Additionally, incentive strategies should be further tested in randomized trials to ascertain causality and confidently determine which strategies are working well and for whom.

## Appendix

Multimedia Appendix 1 STROBE (Strengthening the Reporting of Observational Studies in Epidemiology) checklist.

## Abbreviations

IMD: indices of multiple deprivation
UCL: University College London

## References

[1] Bull FC, Al-Ansari SS, Biddle S, Borodulin K, Buman MP, Cardon G, Carty C, Chaput J, Chastin S, Chou R, Dempsey PC, DiPietro L, Ekelund U, Firth J, Friedenreich CM, Garcia L, Gichu M, Jago R, Katzmarzyk PT, Lambert E, Leitzmann M, Milton K, Ortega FB, Ranasinghe C, Stamatakis E, Tiedemann A, Troiano RP, van der Ploeg HP, Wari V, Willumsen JF (2020). World Health Organization 2020 guidelines on physical activity and sedentary behaviour. Br J Sports Med.

[2] Degenhard J (2024). Number of smarthphone users worldwide 2014-2019. Statistica.

[3] Feter N, Dos Santos Tiago Silva, Caputo EL, da Silva MC (2019). What is the role of smartphones on physical activity promotion? A systematic review and meta-analysis. Int J Public Health.

[4] Mitchell M, Lau E, White L, Faulkner G (2020). Commercial app use linked with sustained physical activity in two Canadian provinces: a 12-month quasi-experimental study. Int J Behav Nutr Phys Act.

[5] Luong MN, Hall M, Bennell KL, Kasza J, Harris A, Hinman RS (2021). The impact of financial incentives on physical activity: a systematic review and meta-analysis. Am J Health Promot.

[6] Hajat C, Hasan A, Subel S, Noach A (2019). The impact of short-term incentives on physical activity in a UK behavioural incentives programme. NPJ Digit Med.

[7] Elliott M, Eck F, Khmelev E, Derlyatka A, Fomenko O (2019). Physical activity behavior change driven by engagement with an incentive-based app: evaluating the impact of Sweatcoin. JMIR Mhealth Uhealth.

[8] Chew L, Tavitian-Exley I, Lim N, Ong A (2021). Can a multi-level intervention approach, combining behavioural disciplines, novel technology and incentives increase physical activity at population-level?. BMC Public Health.

[9] Finkelstein EA, Bilger M, Baid D (2019). Effectiveness and cost-effectiveness of incentives as a tool for prevention of non-communicable diseases: A systematic review. Soc Sci Med.

[10] Rondina R, Hong M, Sarma S, Mitchell M (2021). Is it worth it? Cost-effectiveness analysis of a commercial physical activity app. BMC Public Health.

[11] Charness G, Gneezy U (2009). Incentives to Exercise. Econometrica.

[12] Hunter RF, Gough A, Murray JM, Tang J, Brennan SF, Chrzanowski-Smith OJ, Carlin A, Patterson C, Longo A, Hutchinson G, Prior L, Tully MA, French DP, Adams J, McIntosh E, Xin Y, Kee F (2019). A loyalty scheme to encourage physical activity in office workers: a cluster RCT. Public Health Res.

[13] Washington WD, Banna KM, Gibson AL (2014). Preliminary efficacy of prize-based contingency management to increase activity levels in healthy adults. J Appl Behav Anal.

[14] Wing RR, Jeffery RW, Pronk N, Hellerstedt WL (1996). Effects of a personal trainer and financial incentives on exercise adherence in overweight women in a behavioral weight loss program. Obes Res.

[15] van der Swaluw K, Lambooij MS, Mathijssen JJP, Schipper M, Zeelenberg M, Berkhout S, Polder JJ, Prast HM (2018). Commitment lotteries promote physical activity among overweight adults-a cluster randomized trial. Ann Behav Med.

[16] Petry NM, Andrade LF, Barry D, Byrne S (2013). A randomized study of reinforcing ambulatory exercise in older adults. Psychol Aging.

[17] Patel MS, Volpp KG, Rosin R, Bellamy SL, Small DS, Heuer J, Sproat S, Hyson C, Haff N, Lee SM, Wesby L, Hoffer K, Shuttleworth D, Taylor DH, Hilbert V, Zhu J, Yang L, Wang X, Asch DA (2018). A randomized, controlled trial of lottery-based financial incentives to increase physical activity among overweight and obese adults. Am J Health Promot.

[18] Patel MS, Asch DA, Rosin R, Small DS, Bellamy SL, Heuer J, Sproat S, Hyson C, Haff N, Lee SM, Wesby L, Hoffer K, Shuttleworth D, Taylor DH, Hilbert V, Zhu J, Yang L, Wang X, Volpp KG (2016). Framing financial incentives to increase physical activity among overweight and obese adults: a randomized, controlled trial. Ann Intern Med.

[19] Kullgren JT, Harkins KA, Bellamy SL, Gonzales A, Tao Y, Zhu J, Volpp KG, Asch DA, Heisler M, Karlawish J (2014). A mixed-methods randomized controlled trial of financial incentives and peer networks to promote walking among older adults. Health Educ Behav.

[20] Finkelstein EA, Tham K, Haaland BA, Sahasranaman A (2017). Applying economic incentives to increase effectiveness of an outpatient weight loss program (TRIO) - A randomized controlled trial. Soc Sci Med.

[21] Condliffe S, Işgın E, Fitzgerald B (2017). Get thee to the gym! A field experiment on improving exercise habits. J Behav Exp Econ.

[22] Beatty TK, Katare B (2018). Low-cost approaches to increasing gym attendance. J Health Econ.

[23] Brower J, LaBarge MC, White L, Mitchell MS (2020). Examining responsiveness to an incentive-based mobile health app: longitudinal observational study. J Med Internet Res.

[24] Pearson E, Prapavessis H, Higgins C, Petrella R, White L, Mitchell M (2020). Adding team-based financial incentives to the Carrot Rewards physical activity app increases daily step count on a population scale: a 24-week matched case control study. Int J Behav Nutr Phys Act.

[25] Craig P, Katikireddi SV, Leyland A, Popham F (2017). Natural experiments: an overview of methods, approaches, and contributions to public health intervention research. Annu Rev Public Health.

[26] Bojinov I, Chen A, Liu M (2020). The importance of being causal. Harvard Data Science Review.

