# Supplementary material for: Tracked Physical Activity Levels Before and After a Change in Incentive Strategy Among UK Adults Using a Rewards App: Retrospective Quasi-Experimental Study
Source: JMIR Form Res. 2024 Dec 10;8:e50041. doi: 10.2196/50041 (PMC11668996; doi:10.2196/50041)
Supplement: Multimedia Appendix 1 [file formative_v8i1e50041_app1.docx]

## Appendix

STROBE checklist (Strengthening the Reporting of Observational Studies in Epidemiology)

| **Item No.** | **Checklist Item** | **Reported on Page/Section** |
| --- | --- | --- |
| 1 | Title and Abstract | Title (Page 1), Abstract (Page 1) |
| 2 | Introduction: Background/Rationale | Introduction (Page 2, Paragraphs 2-4) introduction (Page 3, Paragraphs 1&2) |
| 3 | Introduction: Objectives | Introduction (Page 3, Paragraph 3) |
| 4 | Methods: Study Design | Methods (Page 3, Paragraph 4) |
| 5 | Methods: Setting | Methods (Page 3, Paragraph 4) |
| 6 | Methods: Participants | Methods (Pages 4, Paragraphs 1&2) |
| 7 | Methods: Variables | Methods (Page 4, Paragraphs 3,4&5) Methods (Page 5, Paragraphs 1&2) Methods (Page 6, Table 1) |
| 8 | Methods: Data Sources/Measurement | Methods (Page 6, Paragraphs 1&2) Methods (Page 7, Paragraph 1) |
| 9 | Methods: Bias | Methods (Page 7, Paragraph 1) |
| 10 | Methods: Study Size | Methods (Page 7, Paragraph 2) |
| 11 | Methods: Quantitative Variables | NA |
| 12 | Methods: Statistical Methods | Methods (Page 7, Paragraphs 3&4) |
| 13 | Results: Participants | Results (Page 8, Paragraph 1) |
| 14 | Results: Descriptive Data | Results (Page 8, Table 2) |
| 15 | Results: Outcome Data | Results (Page 8, Paragraph 2) Results (Page 9, Table 3) |
| 16 | Results: Main Results | Results (Page 9, Paragraph 1) Results (Page 9, Table 4) |
| 17 | Results: Other Analyses | Results (Page 10, Paragraphs 1&2, Figures 1&2) Results (Page 11, Paragraphs 1, Figure 3) Results (Page 12, Figures 4&5) |
| 18 | Discussion: Key Results | Discussion (Page 13, Paragraphs 1&2) |
| 19 | Discussion: Limitations | Discussion (Page 13, Paragraph 3&4) Discussion (Page 14, Paragraph 1) |
| 20 | Discussion: Interpretation | Discussion (Page 14, Paragraph 3) Discussion (Page 19, Paragraphs 2 & 3 ) |
| 21 | Discussion: Generalizability | Discussion (Page 14, Paragraph 4) |
| 22 | Other Information: Funding | Funding (Page 15, Paragraph 4) |

## 
